# Species presence frequency and diversity in different patch types along an altitudinal gradient: *Larix chinensis* Beissn in Qinling Mountains (China)

**DOI:** 10.7717/peerj.1803

**Published:** 2016-03-15

**Authors:** Minyi Huang, Renyan Duan, Shixiong Wang, Zhigao Wang, Weiyi Fan

**Affiliations:** 1 College of Life Sciences, Anqing Normal University, Anqing, Anhui, China; 2 College of Life Sciences, Shaanxi Normal University, Xi’an, Shaanxi, China; 3 School of Biological and Food Engineering, Suzhou University, Suzhou, Anhui, China

**Keywords:** Patch type, Gap-forming processes, Species diversity, Coexistence, Dispersal limitation

## Abstract

Forest communities are mosaic systems composed of patches classified into four different developmental patch types: gap patch (G), building patch (B), mature patch (M) and degenerate patch (D). To study the mechanisms maintaining diversity in subalpine coniferous forests, species presence frequency and diversity in the four distinct patch types (G, B, M and D) of *Larix chinensis* conifer forests at three altitudinal gradients in the Qinling Mountains were analyzed. Our results were as follows: (1) Different species (or functional groups) had distinct presence frequencies in the four different patch types along the altitudinal gradient; (2) Some species or functional groups (species groups sharing similar traits and responses to the environment) only occurred in some specific patches. For seed dispersal, species using wind mainly occurred in G and D, while species using small animals mainly occurred in B and M; (3) Species composition of adjacent patch types was more similar than non-adjacent patch types, based on the lower *β* diversity index of the former; (4) The maximum numbers of species and two diversity indices (*D*′ and *H*′) were found in the middle altitudes. Various gap-forming processes and dispersal limitation may be the two major mechanisms determining species diversity in *Larix chinensis* coniferous forests at the patch scale.

## Introduction

Over the past century, numerous theories have been proposed to explain species coexistence (Hutchinson, 1957; Paoli, Curran & Zak, 2006; Hubbell, 2001). Prominent among such mechanisms are the niche partitioning theory (Hutchinson, 1957; Paoli, Curran & Zak, 2006) and the unified neutral theory (Hubbell, 2001). The niche partitioning theory (Hutchinson, 1957; Paoli, Curran & Zak, 2006) argues that diversity exists because species partition the environment into unique niches that can vary over time or space, and these niche differences are essential for long-term coexistence. Recently, this mechanism has been challenged by the unified neutral theory (Hubbell, 2001) that assumes species are functionally equivalent without niche differences, and that a series of stochastic events including dispersal limitation are the key factors in maintaining species coexistence. While these two theories undoubtedly contribute to local dynamics underlying coexistence, their relative contributions remain uncertain. Species coexistence is unlikely to be explained by any single theory, and instead several causes likely contribute to understanding the patterns of biodiversity (Chesson, 2000; Ghazoul & Sheil, 2010).

Gaps play an important role in maintaining diversity, including (1) providing colonization sites for shade-intolerant, pioneer species in the community (Silvestrini et al., 2015); (2) partitioning heterogeneous resources, particularly light, for non-pioneer, shade-tolerant species (Westbrook et al., 2011); and (3) creating heterogeneity in understory resources crucial for plant regeneration in closed-canopy forests, allowing plants with contrasting strategies to coexist along resource gradients (Forrester et al., 2014). Most studies on the influence of gaps on species diversity, however, have ignored the development process of gap patch. Patch development processes make a crucial contribution to species composition and biodiversity in a wide variety of forest ecosystems (Galanes & Thomlinson, 2009; Shimatani & Kubota, 2011; Asner et al., 2013; Duan, Huang & Wang, 2013; Král et al., 2014; Blackburn, Abd Latif & Boyd, 2014; Zenner et al., 2015). When disturbances create gaps, suppressed trees and natural regeneration species are released, and a sustained increase in growth is possible as a result of favorable environmental conditions (Blackburn, Abd Latif & Boyd, 2014; Sproull et al., 2015). Natural forests may shift from gaps to a closed canopy, then to gaps again, and then to expanded gap. Gaps in real natural forests usually do not develop into mature types according to a given sequence (Watt, 1947). For various gap-forming processes, canopy dynamics are diverse and patches change with time and space. Diverse patches present heterogeneous environmental factors providing the necessary ecological basis for the coexistence of species (or functional groups) with different ecological demands in their different life history stages (Mejía-Domínguez, Meave & Díaz-Ávalos, 2012). Mosaic patches can further be subdivided: gap and non-gap (Brokaw, 1982); gap, non-gap and expanding gap; gap, closed canopy and gap adjacent areas (Manabe & Yamamoto, 1997); and closed canopy type (CC-Type), gradually closing type (GC-Type), expanding gap (GG-Type) and newly created gap type (NG-Type) (Manabe et al., 2009). A theory of forest dynamics suggests that patch development can be recognized as four patch types: gap patch, building patch, mature patch and degenerate patch (Whitmore, 1989; Zang, Tao & Li, 2005). Further research on changes in species presence, frequency and diversity in the four patch types is needed.

Recently, some studies also document that seed dispersal can determine the species composition of plant communities (Hubbell, 1999; Shen et al., 2009; Hu et al., 2012; Li et al., 2012), distribution (Caughlin et al., 2014), population regeneration (Rey & Alcántara, 2014) and community dynamics (Swamy & Terborgh, 2010). Previous studies show that seed density declines rapidly with distance from the maternal tree (Muller-Landau et al., 2008). Species do not occupy all suitable patches for seed dispersal limitations (Hubbell, 1999; Shen et al., 2009; Wright, 2002; Wang et al., 2013). The available seed pool is not efficient in reaching potential patches for recruitment in natural populations (Hu et al., 2012; Li et al., 2012; Wang et al., 2013). For example, Li et al. (2012) collected seeds for only 52% of species and observed aggregated seed distribution of species among seed traps, which implied that there were strong dispersal limitations in this temperate forest in Northeast China. Knowledge of seed dispersal in a natural forest at the patch scale is valuable for understanding the mechanisms maintaining diversity.

According to the importance of various gap-forming processes and seed dispersal in species composition, we can hypothesize that (1) species (or functional groups) have distinct presence frequencies in distinct types due to their specificity to heterogeneity of habitat patches; (2) various gap-forming processes and seed dispersal limitations can explain the maintenance mechanisms of species diversity within the forest community.

The *Larix chinensis* forest is mainly distributed in elevation from 2,900 to 3,400 m in the Qinling Mountains (Yan, Zhao & Yue, 2000). Natural disturbance intensity varies and includes small-scale disturbances in low altitudes (2,900–3,000 m), large-scale disturbances in high altitudes (3,300–34,200 m), and associated small-scale and large-scale disturbances in middle altitudes (3,100–3,200 m) (Duan et al., 2009). Various disturbance types contribute to diverse gap development processes, forming complicated patch mosaics of four distinct types including gap patch (G), building patch (B), mature patch (M) and degenerate patch (D) (Duan, Huang & Wang, 2013). This offers an opportunity to study the coexistence mechanisms of species diversity. Species presence, frequency and diversity of *L. chinensis* forest among the four distinct patches (G, B, M and D) along an altitudinal gradient were analyzed. The purpose of this study was: (1) to compare the species composition and similarities of different patch types; (2) to identify any differences in species diversity along the altitudinal gradient; and (3) to discuss the maintenance mechanisms of species diversity within the forest community at the patch scale.

## Material and Methods

The Qinling Mountains (32°30′–34°45′N, 104°30′–112°45′E) constitute a huge physical obstacle for the south- and northward movement of air masses due to their east-to-west orientation and high elevation and are thus very important to the distribution of life zones in eastern part of China. The Mt. Taibai (the peak of the Qinling Mountains, 33°57′N, 107°45′E, 3,767 m a.s.l.) is the highest mountain in eastern mainland China. In the Taibai Natural Reserve, the study forests extend from an elevation of 2,900 to 3,400 m on the southern slope. In the study area, *L. chinensis* and *Abies fargesii* are the dominant species; *Betula albo-sinensis*, *Rhododendron capitatum*, *Lonicera webbiana*, *Lonicera hispida*, *Spiraea alpim*, *Salix cupularis*, *Potentilla arbuscula*, *Rosa tsinglingensis* and *Rhododendron clementinae* are the common companion species. Mountain grey-brown forest soil is the main soil type. The climate characteristics are cold winters (average temperature is about −3.6 °C) and wet summers (mean precipitation is about 500 mm). The annual mean temperature and precipitation are 3.4 °C and 910.6 mm, respectively (Tang & Fang, 2006).

Our field studies were granted permission by the Administrative Office of Taibai Natural Reserve to conduct the research there. In 2012, three random sample plots (each 1 ha) were surveyed at low altitude (2,900–3,000 m), mid-altitude (3,100–3,200 m), and high altitude (3,300–3,400 m) in the Taibai Mountain Natural Reserve. In low altitude, the sample is near to tour route, and we can find the traces of human activity, such as some travel rubbishes and the artificial broken branches; while in the middle altitude and high altitude, the sample is far from the tour route and we do not observe the mark of human activity. Every sample plot was divided into 400 grid quadrates (patches) of 5 m × 5 m and there were a total of 1,200 patches of 5 m × 5 m at three altitudes. We investigated whether there were any gap-makers (i.e., branch-broken, trunk-broken, standing dead and uprooted), and whether there were any old individuals. Each patch was numbered and investigated individually. Site factors and general features, such as mean tree height, average height of canopy trees, density, and coverage in *L. chinensis* forest sample plots at three altitudinal gradients are listed in Table 1. The standards for classifying the four patch types (G, B, M and D) and for studying the distribution of patch mosaics and the heterogeneity of light and temperature are provided in a previous publication (Duan, Huang & Wang, 2013).

**Table 1:** Site factors and general features in the *L. chinensis* forest.

Altitude transect (m)	Slope scope (°)	Aspects	Mean tree height (m)	Average height of canopy trees (m)	Density (No.ha^−1^)	Canopy coverage (%)
Low altitude (2,900–3,000)	12–25	S	12.1	16.5	1,695	70
Mid-altitude (3,100–3,200)	8–20	S	6.4	8.6	2,342	41
High altitude (3,300–3,400)	10–28	S	2.8	4.1	817	21

In 2014, to further study diversity and to reduce the effect of density and spatial autocorrelation on plant diversity, 40 patches at each altitudinal gradient in the same forests were chosen (ten of each patch type) according to the following standards: (1) the same patches were not adjacent, and (2) the same patches had similar environment factors (slope scope and aspect) and site factors (density and average height). In total, 120 patches were investigated at three altitudinal gradients. In each patch (5 × 5 m), tree species (diameter at the breast height, *DBH* > 5 cm) were investigated, and four subplots of 2 × 2 m and five subplots of 1 × 1 m were randomly established to investigate shrubs and herbs, respectively. For tree species, the *DBH*, height, numbers and coverage of each individual tree were measured. For the shrub and herbage species, the species names, numbers and coverage were recorded.

For comparing the difference of species diversity and richness in four patches, three indexes: the number of species per unit area (*S*), the Shannon-Wiener diversity index, Eq. (1) (*H*′, Magurran, 2004), and evenness (*J*′, Magurran, 2004) had been used, Eqs. (1) and (2):

(1) \begin{eqnarray*}{H}^{^{\prime}}=-\sum _{i=1}^{s}({P}_{i}\ln {P}_{i})\end{eqnarray*}

(2) \begin{eqnarray*}{J}^{^{\prime}}={H}^{^{\prime}}/\ln S\end{eqnarray*}

where *H*′ is the Shannon–Wiener index and *J*′ is the Pielou Evenness index. *P*_*i*_ = *N*_*i*_∕*N*, *N*_*i*_ is the number of species *i* and *N* isthe sum of all species found in each layer of stand.

For comparing and distinguishing the difference of species compositions among four different patch types at the three altitudes, the *β* diversity index was used, Eq. (3) (Legendre & Legendre, 1998):

(3) \begin{eqnarray*}CD=1-2c/(a+b)\end{eqnarray*}

where *CD* is species shared in both forest types and sample sites; *a* and *b* are species existing only in one patch; and *c* is species existing in both patches.

Functional groups are species groups sharing similar traits and response to environment sharing similar traits and response to environment (Tilman et al., 1997). Species and functional diversity are correlated; each was significant by itself, as was species diversity within functional groups. Measuring functional groups may provide a useful gauge of species or functional diversity (Tilman et al., 1997). Plant functional types can be defined as seed dispersal types and life form. Life forms are classified as annual grasses, perennial grasses, ferns, shrubs, and trees (Nagaike, Kamitani & Nakashizuka, 1999). In our study area, there are no vine species. In order to examine whether species occurred disproportionately in different patch types in different altitudes, each species was classified into six different patch groups: gap type (GS), building type (BS), mature type (MS), degeneration type (DS), not biased (generalist species, GES), and too infrequent for statistical analysis (infrequent species, INF), according to whether they appear disproportionately in special patches. The presence frequency differences of all species found in particular patch types were tested statistically using the chi-square test and Fisher’s exact test based on the procedure of Nagaike, Kamitani & Nakashizuka (1999). In regard to seed dispersal mechanisms, plants can be classified into three main types, including wind, birds, and small animals (e.g., ants, rodents) (Nagaike et al., 2006). If species have several dispersal modes, those with fleshy fruits were classified as bird-dispersed and those with nuts as small animal-dispersed (Nagaike et al., 2006).

Here, we mainly focused on the key effect of various gap-forming processes and dispersal limitation without considering the effect of other site factors (e.g., soil, land form and slope direction). We used one-way ANOVA analysis in STATISTICA 7.0 (StatSoft, Inc.; Tulsa, USA). Significant differences discussed had a probability (*P*) value <0.05. If the data did not follow a normal distribution or homogeneity of variance, they were analyzed with the Kruskal–Wallis test, instead of parametric ANOVA.

## Results

The presence frequencies of main woody plants showed significant differences among different patch types (*p* < 0.05) (Table 2). Some species only existed in certain phases of forest patches. For example, *Lonicera hispida* only existed in G at low and middle altitudes (Table 2), while other species were present in all types. Some species only occurred in certain altitudes. For example, *Rosa tsinglingensis* were found only in low altitudes (Table 2), while *Spiraea alpim* and *Salix cupularis* were found only in high altitudes (Table 2). Even the dominant species, such as *L. chinensis* and *A. fargesii*, showed different presence frequencies. For example, *L. chinensis* showed the highest presence frequency in G, while *A. fargesii* had the lowest presence frequency in the same patch (Table 2).

**Table 2:** The presence frequencies (%) of ten main woody plants in different patch types of the forest cycle in the *L. chinensis* forest. For each comparison, different letters indicate means with a significant difference (LSD test, *P* < 0.05, *a* > *b* > *c* > *d*) between different patch types (G, B, M and D) at the same altitude.

Species	Patch	Low altitude	Mid-altitude	High altitude
*Larix chinensis*	G	83.3 ± 7.6 a	93.1 ± 12.9 a	96.1 ± 9.5 a
	B	48.2 ± 9.2 c	75.1 ± 10.5 b	64.1 ± 15.3 b
	M	64.1 ± 8.9 b	90.4 ± 14.3 a	93.8 ± 9.6 a
	D	79.2 ± 9.8 a	91.8 ± 7.2 a	90.7 ± 13.2 a
*Abies fargesii*	G	44.6 ± 5.2 c	18.5 ± 8.3 c	–
	B	72.8 ± 8.1 b	42.4 ± 7.3 b	–
	M	82.1 ± 14.1 a	70.9 ± 8.2 a	–
	D	50.1 ± 6.3 c	47.8 ± 7.6 b	–
*Betula albosinensis*	G	68.2 ± 13.9 a	19.2 ± 3.7 a	–
	B	6.3 ± 2.6 c	8.8 ± 5.1 bc	–
	M	8.1 ± 2.3 c	6.2 ± 1.9 c	–
	D	16.5 ± 7.3 b	10.3 ± 5.2 b	–
*Lonicera webbiana*	G	46.3 ± 6.2 a	34.3 ± 8.9 a	–
	B	23.5 ± 5.1 b	28.2 ± 7.4 a	–
	M	45.9 ± 4.7 a	5.7 ± 3.2 c	–
	D	43.6 ± 8.9 a	15.1 ± 2.1 b	–
*Lonicera hispida*	G	12.4 ± 6.5	16.5 ± 8.7	–
	B	–	–	–
	M	–	–	–
	D	–	–	–
*Spiraea alpim*	G	–	–	18.1 ± 4.2 a
	B	–	–	9.2 ± 3.6 b
	M	–	–	4.4 ± 1.7 c
	D	–	–	16.5 ± 5.5 a
*Salix cupularis*	G	–	–	56.3 ± 7.6 a
	B	–	–	15.2 ± 6.7 c
	M	–	–	8.3 ± 2.9 d
	D	–	–	40.9 ± 3.1 b
*Rhododendron capitatum*	G	36.2 ± 6.1 a	48.1 ± 8.0 a	74.7 ± 9.8 a
	B	10.1 ± 3.7 b	39.8 ± 5.6 a	70.5 ± 6.9 a
	M	–	3.1 ± 2.9 b	39.2 ± 6.4 b
	D	–	9.3 ± 4.5 b	48.7 ± 9.1 b
*Potentilla arbuscula*	G	33.3 ± 9.1 a	27.2 ± 8.1 a	45.7 ± 5.2 a
	B	18.0 ± 9.8 bc	18.2 ± 6.4 b	16.8 ± 6.0 c
	M	10.9 ± 6.6 c	21.1 ± 7.8 ab	20.5 ± 11.2 c
	D	21.7 ± 7.0 b	23.7 ± 6.6 ab	31.9 ± 10.9 b
*Rosa tsinglingensis*	G	10.7 ± 4.4 a	–	–
	B	9.1 ± 3.0 a	–	–
	M	5.7 ± 2.9 b	–	–
	D	8.3 ± 4.8 ab	–	–

**Notes.**

G Gap patch B Building patch M Mature patch D Degenerate patch

**Figure 1:**
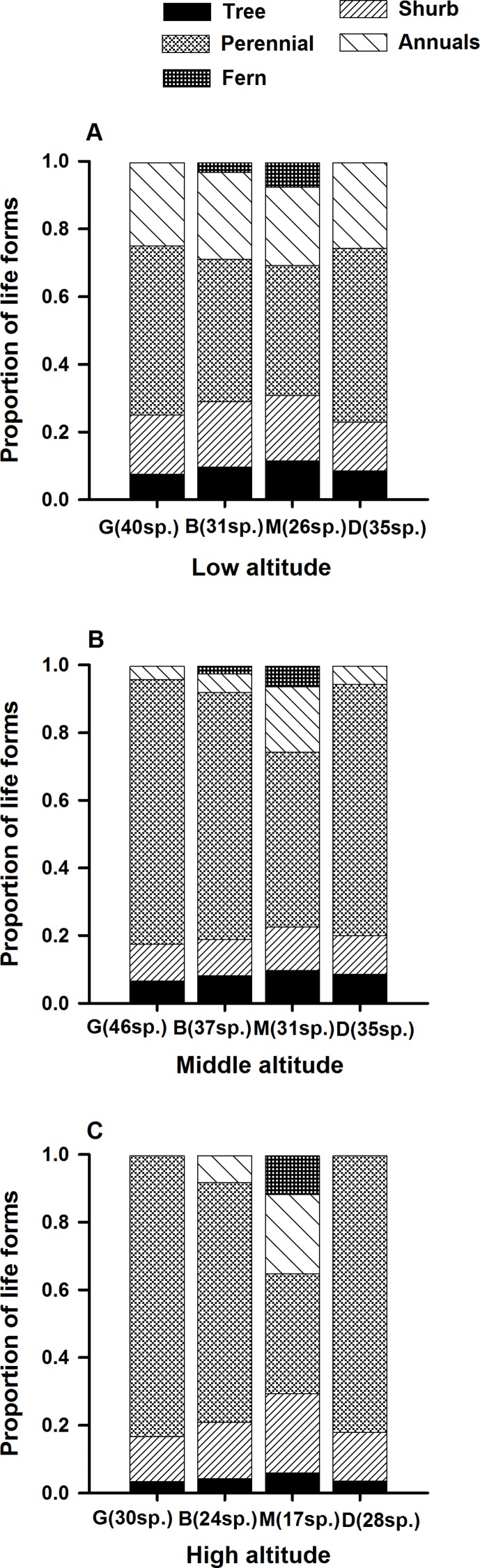
Changes in species compositional proportion of plant life forms in the *L. chinensis* forest. G, gap patch; B, building patch, M, mature patch; D, degenerate patch.

**Figure 2:**
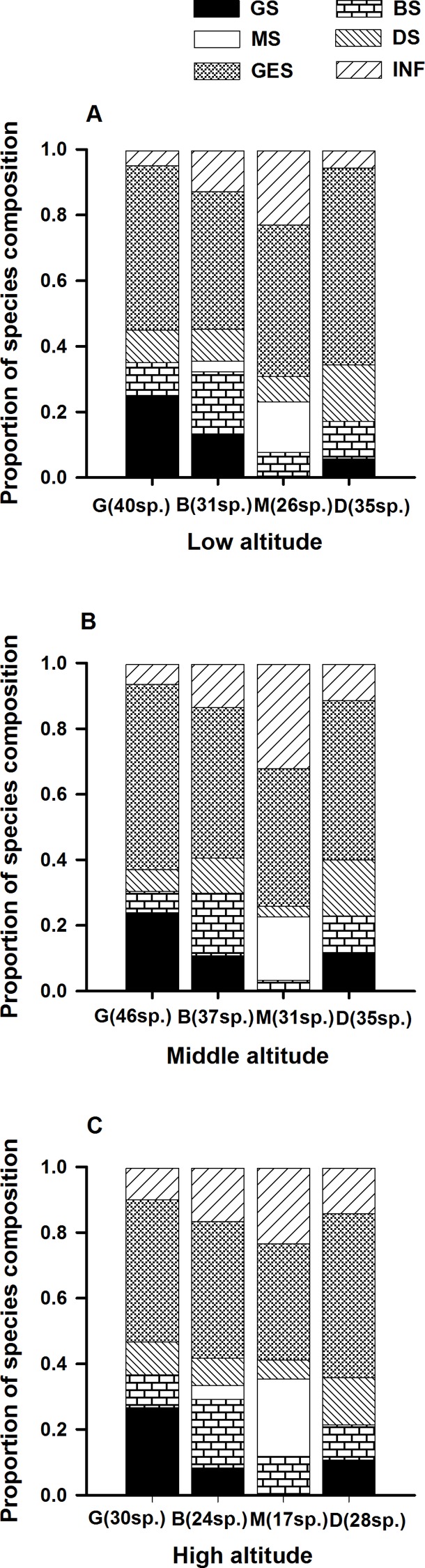
Changes of species compositional proportion of functional groups in the *L. chinensis* forest. G, gap patch; B, building patch; M, mature patch; D, degenerate patch. GS, species mainly present in gap patches; BS, species mainly present in building patches; MS, species mainly present in mature patches; DS, species mainly present in degeneration patches; GES, generalist species; INF, infrequent species.

**Figure 3:**
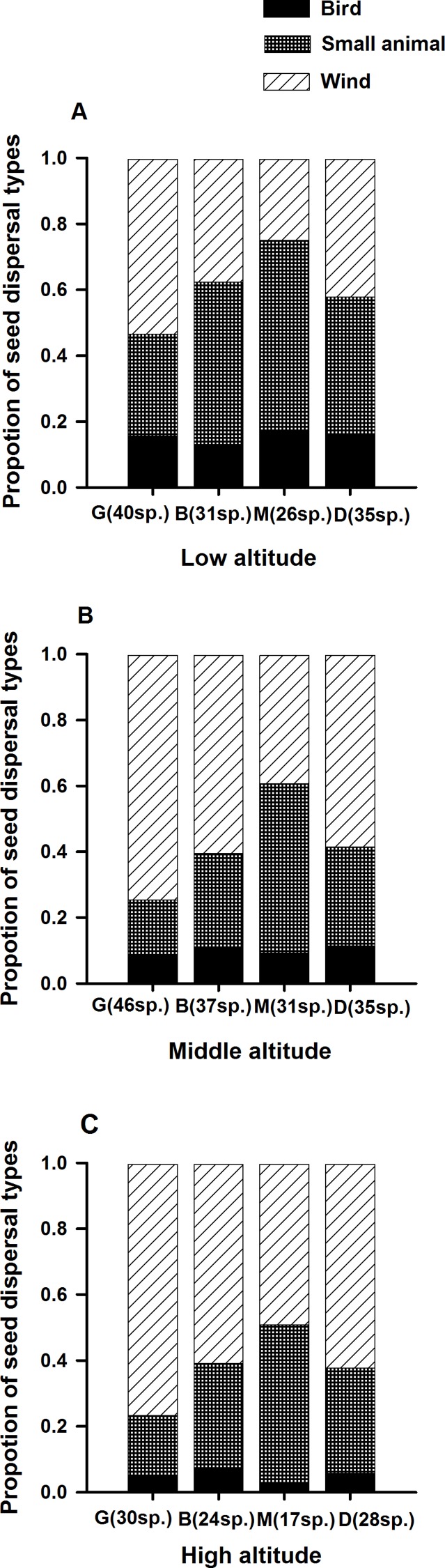
Species seed dispersal types in *L. chinensis* forest. G, gap patch; B, building patch; M, mature patch; D, degenerate patch.

At the three altitudinal gradients, the proportion of life form type among species in different patches varied (Figs. 1–3). Ferns only occurred in B and M (Fig. 1), while fewer GS and more INF species were found in M (Fig. 2). Species using wind for seed dispersal mainly occurred in G and D, and species using small animals for seed dispersal mainly occurred in B and M. Species using birds showed no significant difference among the four patch types (Fig. 3).

**Figure 4:**
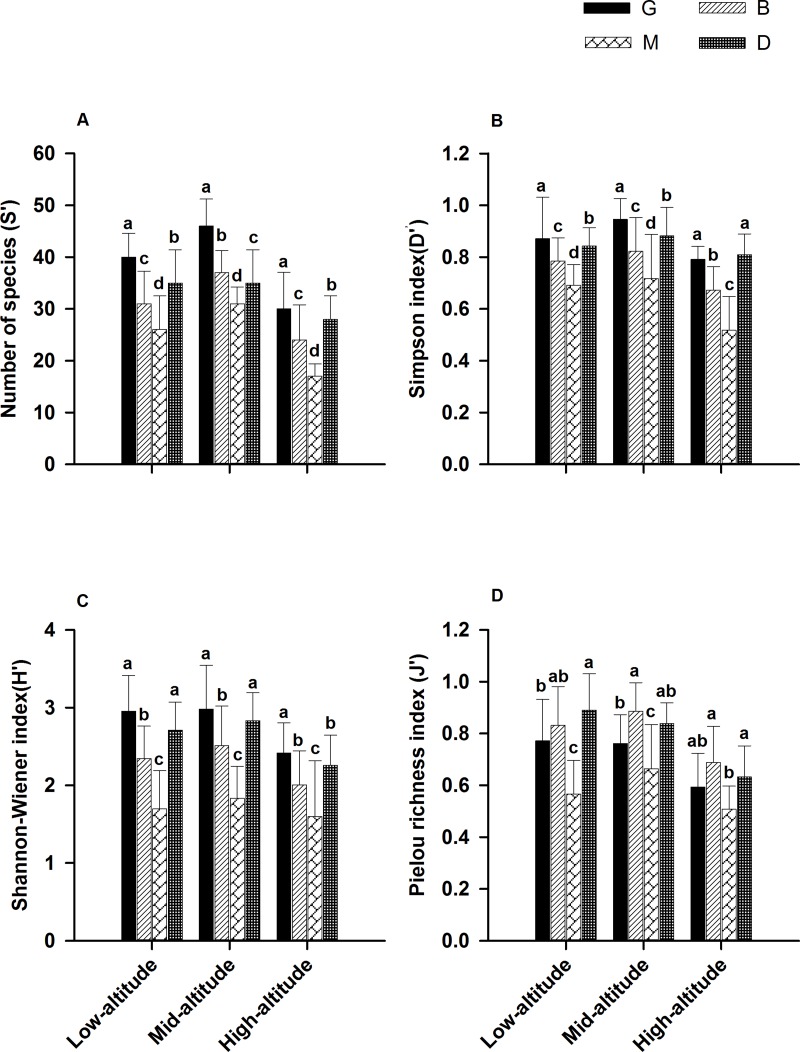
Species diversity indices for species in different patch types of the forest cycle in the *L. chinensis* forest. For each comparison, different letters indicate mean with a significant difference (LSD test, *P* < 0.05, *a* > *b* > *c* > *d*) between different patch types (G, B, M and D) at the same altitude. G, gap patch; B, building patch; M, mature patch; D, degenerate patch.

In the three distribution altitudes, the number of species, diversity indices (*D*′ and *H*′) and evenness index (*J*′) at the middle altitudes showed the highest values (Fig. 4). Species diversity showed a humpback model in the forest cycle process (Fig. 4).

The analysis of the *β* diversity index also indicated large differences between different patch phases (Table 3). In the tree and shrub layer, species composition was almost the same between different patch types, while in the herb layer and the total, composition differed significantly between different patch types. In the herb layer and the total, the *β* diversity index of two adjacent patch types (G–B, B–M and M–D) was lower than that of non-adjacent patch types (G–M, G–D and B–D) (Table 3). For example, in the herb layer, the *β* diversity index between G and B was lower than that of the gap type and mature type, indicating that species composition was more similar at adjacent than at non-adjacent patch types.

**Table 3:** *β* diversity index in different patch types of the forest cycle in the *L. chinensis* forest.

	Patch	Tree layer			Shrub layer			Herb layer			Total		
		B	M	D	B	M	D	B	M	D	B	M	D
Low altitude	G	0	0	0	0.08	0.17	0.17	0.35	0.58	0.16	0.27	0.45	0.15
	B		0	0		0.09	0.09		0.35	0.39		0.26	0.30
	M			0			0			0.64			0.48
Mid-altitude	G	0	0	0	0.11	0.11	0.11	0.32	0.65	0.30	0.28	0.53	0.26
	B		0	0		0	0		0.52	0.10		0.41	0.08
	M			0			0			0.54			0.42
High altitude	G	0	0	0	0	0	0	0.32	0.62	0.17	0.26	0.49	0.14
	B		0	0		0	0		0.42	0.24		0.32	0.19
	M			0			0			0.54			0.42

**Notes.**

G Gap patch B Building patch M Mature patch D Degenerate patch

## Discussion

At the three altitude ranges, there are various interference modes. At low altitudes, small-scale interferences (e.g., interspecific competition, intraspecific competition and human activities) dominate and in high altitudes, there large-scale interferences (e.g., snowstorm, strong wind) are more prominent. In middle altitudes, the greatest variability in interference types is found, including some small-scale interferences and some large-scale interferences (Duan et al., 2009). Various interference types lead to high species diversity indices, which may be the main reason for the highest diversity indices at the middle altitudes, compared with low and high altitudes.

Various gap interferences change patch size and development, and cause habitat heterogeneity, leading to the different presence frequencies of species (Manabe et al., 2009; Blackburn, Abd Latif & Boyd, 2014; Král et al., 2014). When these disturbances are continuous (e.g., gap expansions), the series of the events (gap-forming process) become quite diverse (Manabe et al., 2009; Galanes & Thomlinson, 2009). Various progresses of gap formation and development form a complicated patch mosaic (Manabe et al., 2009). Our previous study documented the mosaic-complex forest community in this subalpine coniferous forest and found diverse patch distribution percentages and distribution patterns at three different altitudinal gradients (Duan, Huang & Wang, 2013). This forest community is composed of a series of small, shifting mosaic patches of various types. We also observed the gap development processes were various. For example, (1) twelve adjacent patches in low altitude studied were in G patch in 2006, but they were in different patch types (three B patches, five M patches, two D patches and two G patches) in 2014; and (2) the patches with adjacent developments (such as G–B, B–M and M–D) were not always adjacent. For example, not only B patches but also other patch types (such as the M and D patches) were around the G patch (Duan, Huang & Wang, 2013), which may indicate the gap development processes is various. Diverse gap development processes indicate that the old-growth subalpine coniferous forest is not a simple aggregation with different patches. Each patch has experienced different disturbance regimes with various spatial scales (per unit area) and frequencies (per unit time), and thus each patch has a diverse stand structure.

Diverse gap development processes relying on small-scale disturbances form the complicated patch mosaic, which is one of the main factors in maintaining the heterogeneous microenvironment in different patches. However, there are limited micro-environmental conditions and topographic diversity in every 1-ha subalpine coniferous forest stand. Our earlier study on the same forest demonstrated that different patches have a heterogeneous microenvironment, including differences in light, air temperature and soil temperature (Duan, Huang & Wang, 2013). These heterogeneous environmental factors can play an important role in the growth and regeneration of plants in different patch types with different ecological characteristics. Heterogeneous patches show selectivity for species of a diversity sizes and functions (Mejía-Domínguez, Meave & Díaz-Ávalos, 2012). Our present results support and enrich those of previous studies. For example, we observed that species frequency and composition, and functional group composition in different patches at the three altitudinal gradients were significantly different. The different species diversity among the four patch types (G, B, M and D) may be a result of a micro-environmental heterogeneity, both vertically and horizontally, created by diverse gap development processes. These results indicate that these diverse gap development processes are beneficial for the maintenance of species diversity in subalpine coniferous forests.

Species diversity in different patches can also be affected by species dispersal limitations (Hu et al., 2012; Hubbell, 1999; Ehrlen & Eriksson, 2000). We observed that diversity in the same developmental stage had a larger standard deviation in every sample and a significant difference among different samples, although patches at the same developmental stage had a similar micro-environment. Our results indicate that dispersal limitation may play a key role. Because of dispersal limitations, available seeds are not efficient in arriving at potential patches for regeneration and recruitment in natural populations, and species can not occupy all suitable patches (Hu et al., 2012). We also observed that some species, such as *L. hispida*, fern, and INF species, only occur in particular habitats. Interestingly, a large number of these species can be found in all patches near our study site. These results indicate that establishment and regeneration of plants may fail for reasons other than patch unsuitability. We presume this may be caused by dispersal limitations rather than species specificity for patches. Other studies support this idea. For example, Ehrlen & Eriksson (2000) suggest that species do not exhibit higher emergence, establishment and survival in their occupied suitable patches compared to unoccupied patches, regarding patches in which at least one seedling survived to the third year in 48 patches in seven temperate forest herbs. In our study, the dominant tree species, *A. fargesii* (a shade-tolerant species) and *L. chinensis* (a light-demanding species), can occur in all the patches, but field surveys suggested that saplings of *A. fargesii* were present in all patches and the seedlings of *L. chinensis* only occurred in the gap type.

Species with different regeneration characteristics, whose seedlings may be found in a specific microenvironment, can share continuous canopy layer space in the adult stage, adding species diversity in the community (Manabe et al., 2009; Forrester et al., 2014; Blackburn, Abd Latif & Boyd, 2014). In our study, the similarities of species compositions in non-neighboring and neighboring patch types are higher than for non-neighboring patch types, which implies that most patches involve gradual change, and there exists a certain dispersal limitation among non-neighboring patch types. Recently, many empirical and theoretical studies have provided strong support for the hypothesis that dispersal limitation plays a critical role in the maintenance of species diversity in community processes (Galanes & Thomlinson, 2009; Hubbell, 1999; Shen et al., 2009; Hu et al., 2012; Li et al., 2012; Wang et al., 2013). For example, dispersal limitation has been reported as the main factor controlling tree species diversity at early patch stages in a northern edge of the Asian tropical rain forests in China (Hu et al., 2012). Some studies suggest that the spatial distribution is affected by seed dispersal, germination and the formation of sapling banks (Manabe et al., 2009; Li et al., 2012; Wang et al., 2013). These studies on dispersal limitation mainly come from a large scale (>25 ha) and/or long-time (>3a) dynamic research, while our research documented that there was seed dispersal limitation at a small scale (1 ha), which provided additional evidence in a small scale about diffusion restriction theory.

## Conclusions

*Larix chinensis* conifer forests can be divided into four patch types: gap patch (G), building patch (B), mature patch (M) and degenerate patch (D). Species composition, presence frequency and diversity differed not only in different patches, but also in the same patches in the forests. Diverse gap-forming processes relying on natural disturbances form the complicated patch mosaic, which may be the main factors in maintaining the diversity in different patches, while seed dispersal limitations play a key role in maintaining the diversity in the same patches. Various gap-forming processes caused by various disturbance modes and seed dispersal limitations can determine species composition and coexistence at the patch scale.

## Supplemental Information

10.7717/peerj.1803/supp-1 Table S1 Species in the Larix chinensis forest

10.7717/peerj.1803/supp-2 Data S1 Raw data

## Additional Information and Declarations

### Field Study Permissions

The following information was supplied relating to field study approvals (i.e., approving body and any reference numbers): Our field studies were granted permission by the Administrative Office of the Taibai Natural Reserve to conduct the research there (permit number (20120810)).

### Data Availability

The following information was supplied regarding data availability: The raw data has been supplied as Data S1.
