# Supplementary material for: Species presence frequency and diversity in different patch types along an altitudinal gradient: Larix chinensis Beissn in Qinling Mountains (China)
Source: PeerJ. 2016 Mar 15;4:e1803. doi: 10.7717/peerj.1803 (PMC4797764; doi:10.7717/peerj.1803)
Supplement: Table S1 [file peerj-04-1803-s001.doc]

**Table S1 Species in the *L. chinensis* forest**

| Species | Family | Altitude | Life form |
| --- | --- | --- | --- |
| *Betula albosinensis* | Betulaceae | low, middle | Tree |
| *Lithospermum zollinged* | Boragmaceae | low | Herb |
| *Myosotis caespitosa* | Boragmaceae | middle | Herb |
| *Myosotis silvatica* | Boragmaceae | low, middle | Herb |
| *Trigonotis giraldii* | Boragmaceae | low | Herb |
| *Trigonotis mollis* | Boragmaceae | low | Herb |
| *Adenophora paniculata* | Campanulaceae | low, middle | Herb |
| *Adenophora potaninii* | Campanulaceae | low, middle, high | Herb |
| *Adenophora stricta* | Campanulaceae | middle | Herb |
| *Adenophora petiolata* | Campanulaceae | low, middle | Herb |
| *Lonicera hispida* | Caprifoliaceae | low, middle | Shrub |
| *Lonicera webbiana* | Caprifoliaceae | low, middle | Shrub |
| *Cerastium arvense* | Caryophyllaeeae | high | Herb |
| *Cerastium caespitosum* | Caryophyllaeeae | middle, high | Herb |
| *Cerastium furfatum* | Caryophyllaeeae | low, middle, high | Herb |
| *Melandrium apetalum* | Caryophyllaeeae | low, middle, high | Herb |
| *Pscudostcllaria heterophylla* | Caryophyllaccae | middle | Herb |
| *Silene fortunei* | Caryophyllaeeae | middle, high | Herb |
| *Silene tenuis* | Caryophyllaeeae | middle, high | Herb |
| *Stellaria alsine* | Caryophyllaceae | low, middle | Herb |
| *Seneeio winklerianus* | Compositae | middle | Herb |
| *Ajania potaninii* | Compositae | high | Herb |
| *Ajania potaninii* | Compositae | middle, high | Herb |
| *Anaphalis aumopunctata* | Compositae | low, middle, high | Herb |
| *Anaphalis flavescens* | Compositae | low, middle, high | Herb |
| *Anaphalis margaritacea* | Compositae | low, middle | Herb |
| *Aster albescens* | Compositae | high | Herb |
| *Aster alpinus* | Compositae | middle, high | Herb |
| *Ligularia purdomii* | Compositae | high | Herb |
| *Saussurea cauloptera* | Compositae | middle | Herb |
| *Saussurea purpurascens* | Compositae | low, middle, high | Herb |
| *Saussurea sobarocephala* | Compositae | low, middle, high | Herb |
| *Sonchus oleraceus* | Compositae | middle, high | Herb |
| *Taraxacum borealisinense* | Compositae | high | Herb |
| *Rhodiola eurycarpa* | Crassulaceae | low, middle | Herb |
| *Rhodiola kirilowii* | Crassulaceae | low, middle | Herb |
| *Sedum pampaninii* | Crassulaceae | low, middle | Herb |
| *Sedum chauveaudiiRaym* | Crassulaceae | low, middle | Herb |
| *Arabis pendula* | Cruciferae | high | Herb |
| *Braya heterophylla* | Cruciferae | middle, high | Herb |
| *Cardamine macrophylla* | Cruciferae | low, middle, high | Herb |
| *Cardamine stenoloba* | Cruciferae | low, middle | Herb |
| *Draba ladyginii* | Cruciferae | low, middle, high | Herb |
| *Carex capilliformis* | Cyperaceae | low, middle, high | Herb |
| *Carex eapilliformis* | Cyperaceae | middle | Herb |
| *Carex filamentosa* | Cyperaceae | low, middle | Herb |
| *Carex kansuensis* | Cyperaceae | middle, high | Herb |
| *Carex kwangtoushanica* | Cyperaceae | middle, high | Herb |
| *Carex lehmannii* | Cyperaeeae | low, middle, high | Herb |
| *Carex ligulata* | Cyperaceae | low, middle | Herb |
| *Carex longerostrata* | Cyperaceae | middle | Herb |
| *Carex luctuosa* | Cyperaceae | low, middle | Herb |
| *Carex rochebruni* | Cyperaceae | middle, high | Herb |
| *Carex scabrirostris* | Cyperaceae | middle, high | Herb |
| *Carex schneideri* | Cyperaceae | middle | Herb |
| *Kobresia graminifolia* | Cyperaceae | low, middle, high | Herb |
| *Kobresia prattii* | Cyperaceae | low, middle, high | Herb |
| *Rhododendron capitatum* | Ericaceae | low, middle, high | Shrub |
| *Indigofera bungeana* | Leguminosae | middle | Herb |
| *Gentiana apiata* | Gentianaceae | high | Herb |
| *Gentiana crassuloides* | Gentianaceae | middle | Herb |
| *Gentiana flexicaulis* | Gentianaceae | middle | Herb |
| *Gentiana hexaphylla* | Gentianaceae | middle | Herb |
| *Gentiana rhodamha* | Gentianaceae | middle | Herb |
| *Lonatogoniumperenne* | Gentianaceae | high | Herb |
| *Swertoa bifolia* | Genfianaceae | high | Herb |
| *Deschampsia caespiusa* | Genfianaceae | low, middle, high | Herb |
| *Deyeuxia conferto* | Genfianaceae | middle | Herb |
| *Deyeuxia hupehensis* | Gramineae | middle, high | Herb |
| *Festuca ovina* | Gramlneae | low, middle, high | Herb |
| *Poa aflllua* | Gramineae | low, middle | Herb |
| *Poa angustifolia* | Gramineae | middle | Herb |
| *Poa ncmoralis* | Gramineae | low, middle, high | Herb |
| *Poa otinosa* | Gramineae | low, middle, high | Herb |
| *Poa sirmttcnuata* | Gramineae | middle, high | Herb |
| *Proegneria kamoji* | Gramineae | middle, high | Herb |
| *Ptilagrostis hispidus* | Gramineae | low, middle, high | Herb |
| *Trisctum spieatum* | Gramineae | low, middle | Herb |
| *Trisetum spicatum* | Gramineae | middle, high | Herb |
| *Juncus allioides* | Juglandaceae | middle, high | Herb |
| *Juncus modicus* | Juncaceae | middle, high | Herb |
| *Juncus przcwalskii* | Juncaccae | low, middle, high | Herb |
| *Gueldenstaedtia multiflora* | Legummosae | low | Herb |
| *Hedy sarumtaipeicum* | Legummosae | middle, high | Herb |
| *Oxytropis ehinglingensis* | Legummosae | low, middle, high | Herb |
| *Allium maeranthum* | Liliaeeae | low, middle, high | Herb |
| *Allium prattii* | Liliaeeae | middle, high | Herb |
| *AIlium sikkimense* | Liliaeeae | middle, high | Herb |
| *Notholirion hyacinthlnum* | Liliaceae | low, middle | Herb |
| *Polygonatum verticillatum* | Liliaceae | low, middle | Herb |
| *Vcratram nigrum* | Liliaceae | low, middle | Herb |
| *Alctris glabra* | Liliaceae | middle | Herb |
| *Epilobium palustre* | Onagraceae | high | Herb |
| *Epilobium parviflorum* | Onagraceae | middle, high | Herb |
| *Oxalis acetosella* | Oxalidaceae | low, middle | Herb |
| *Corydalis trisecta* | Fumariaceae | low, middle, high | Herb |
| *Corydalis curviflora* | Fumariaceae | low, middle, high | Herb |
| *Meconopsis oliverana* | Fumariaceae | middle | Herb |
| *Meconopsis quintuplinercia* | Fumariaceae | low, middle, high | Herb |
| *Polygonum bubertii* | Polygonaceae | middle, high | Herb |
| *Polygonum sphoerostachyum* | Polygonaceae | low, middle, high | Herb |
| *Polygonum viviparum* | Polygonaceae | low, middle | Herb |
| *Primula conspersa* | Primulaceae | high | Herb |
| *Primula moupinensis* | Primulaceae | low, middle, high | Herb |
| *Primula purdomii* | Primulaceae | low, middle, high | Herb |
| *Aconitum szcchenyianum* | Rantmculaceae | low, middle | Herb |
| *Aconitum taipaicum* | Rantmculaceae | low, middle, high | Herb |
| *Aconimm tanguticum* | Rantmculaceae | high | Herb |
| *Anomono taipaiensis* | Rammculaceae | low, middle, high | Herb |
| *trollius farrei* | Ranunculaceae | low, middle, high | Herb |
| *Fragaria graeilis* | Rosaeeae | low, middle | Herb |
| *Potentilla arbuseulla* | Rosaeeae | low, middle, high | Shrub |
| *Rosa tsinglingensis* | Rosaeeae | low | Shrub |
| *Potentilla erioearpa* | Rosaeeae | middle | Herb |
| *Potentilla sino-nivea* | Rosaeeae | middle, high | Herb |
| *Spiraea alpim* | Rosaceae | high | Shrub |
| *Galiu bungei* | Rubiaecae | low, middle | Herb |
| *Saxifraga genunigera* | Saxifragaceae | low, middle, high | Herb |
| *Saxifraga giraldiana* | Saxifragaceae | low, middle, high | Herb |
| *Saxifraga melanocentra* | Saxifragaceae | low, middle, high | Herb |
| *Saxifraga montana* | Saxifragaceae | low, middle, high | Herb |
| *Chrysosplenium griffithii* | Saxifragaceae | low, middle, high | Herb |
| *Parnassia viridiflom* | Saxifragaceae | low, high | Herb |
| *Pedicularis docora* | Scrophulariaceae | low, middle, high | Herb |
| *Pedicularis dissecta* | Scrophulariaceae | middle, high | Herb |
| *Veronica ciliata* | Scrophulariaccac | low, middle, high | Herb |
| *Salix cupularis* | Salicaceae | high | Shrub |
| *Pleurospermum giraldii* | Umbelliferae | low, middle, high | Herb |
| *Abics fargesii* | Pinaceae | low, middle | Tree |
| *Larix chinensis* | Pinaceae | low, middle, high | Tree |
| *Cryptogramma raddeana* | Sinopteridaceae | low, middle, high | Herb (Fern) |
| *Adiantum fimbriatum* | Adiantaceae | low, middle, high | Herb (Fern) |
| *Woodsia polystichoides* | Woodsiaceae | low, middle | Herb (Fern) |
